# Perspectives on peer-review and editorial activities of Peruvian dental researchers

**DOI:** 10.21142/2523-2754-1104-2023-170

**Published:** 2023-12-28

**Authors:** Angela Quispe-Salcedo

**Affiliations:** 1 Division of Anatomy and Cell Biology of the Hard Tissue. Niigata University Graduate School of Medical and Dental Sciences, Niigata, Japan. aquispesa@dent.niigata-u.ac.jp Division of Anatomy and Cell Biology of the Hard Tissue Niigata University Graduate School of Medical and Dental Sciences Niigata Japan aquispesa@dent.niigata-u.ac.jp

Since the establishment of new strategies for the promotion of academic research activity in public and private higher education institutions, driven by the declaration of Peruvian University Law 30220 in 2014, scientific production in dental research have progressively increased in our country. As shown by a recent bibliographic study on the scientific production of Peruvian Dental Schools from 2014 to 2019 after the enactment of this law, all public and private schools notably increased their contributions, being three institutions: Peruvian University Cayetano Heredia- School of Dentistry, National University of San Marcos Faculty of Dentistry, and Cientifica del Sur University School of Stomatology) the highest scoring in the SCOPUS scientific database ^1^.

Although there is no data available on these matters, it is presumed that these numbers have been pushed by initiative of Peruvian early-career dental researchers with -or without- support of mid-career researchers already established in the country. While the younger generation is learning how to develop a project and write the manuscripts, mid-career researchers may be mentoring these initiatives and, together, learning to navigate the oceans of peer-reviewing process in international academic journals with impact factor. The present scenario is definitely encouraging for future generations of researchers, as the foundations for future academic activity have already been established. However, there is another side of research activity that should be encouraged among our early- and mid-career dental researchers: their participation in editorial and peer-review activities.

To participate in a peer-review process, it is necessary for the researcher to have acquired some experience in certain fields of research, including technical and substantive knowledge. Also, being a reviewer is by no means an easy task; it is time consuming, and contrary to what some believe, the activity of a reviewer of any journal is not recognized or remunerated. However, the process itself its highly instructive as it can allow us to know the reader’s viewpoint. It brings the opportunity to learn the pitfalls of the studies and the effective strategies used by other researchers, to improve our writing, and broaden the perspective of our field of expertise. No researcher has started this journey knowing how to make a good judgement on the work of another, but these criteria can be obtained through repetition and self-learning from currently available sources aimed to enhance the reviewer’s role. It is therefore important that mentors can encourage Peruvian dental graduate students and early-career dental researchers to serve as reviewers for local dental journals, matching their expertise to the level of rigor of the publication ^2^^,^^3^.

Another important aspect to be improved is the participation of Peruvian dental researchers of any career level in the editorial activity. Being a member of the board of a dental journal in our country is s one of the least encouraged roles among academics. In fact, the lack of indexed dental journals, even in local databases, has created a void in the path to become a journal editor. While it is true that the management of Peruvian Dental School Journals is in charge of faculty members, there are tasks in the process to complete a Journal’s volume that can be offered to young researchers to help them to gain editorial experience. Another chance is given by dental societies journals, where usually the roles of editor in chief and associate editor are changing more often that School Journals, and they are always in need of people who wants to strive for the development of these publications. In addition, other good points to consider are the chances to expand the professional network who may provide new career opportunities, and the access to insider information on what topics can be published or not ^4^^,^^5^.

Dental research is complex but must be known in depth in all its areas, from the point of view of those who develop research and manage to publish it, as well as those who evaluate and manage the contents of a scientific journal. It is important that the new generation of Peruvian dental researchers acquire the necessary experience for generational change in both aspects. Our local journals are just taking off, and surely in the near future, their scientific quality will meet international indexing standards. Therefore, mentors and early-career researchers have to be prepared to take the post and face the challenge.

## References

[1] Mayta-Tovalino F, Pacheco-Mendoza J, Díaz-Soriano A, Pérez-Vargas F, Munive-Degregori A, Luza S (2021). Bibliometric Study of the National Scientific Production of All Peruvian Schools of Dentistry in Scopus. Int J Dent.

[2] Morley CP, Grammer S (2021). Now More Than Ever: Reflections on the State and Importance of Peer Review. PRiMER.

[3] Kerig PK (2021). Why Participate in Peer Review?. J Trauma Stress.

[4] D'Agostino S (2019). Why you should join a journal's editorial board. Nature.

[5] Menon V (2023). Serving as a Handling Editor? Thirteen Simple Messages for Early-Career Editors. Indian J Psychol Med.

